# High rates of anorectal chlamydia in women: a cross-sectional study in general practice

**DOI:** 10.3399/BJGPO.2021.0223

**Published:** 2022-06-29

**Authors:** Elisabeth AB, Dirk Luijt, Alewijn Ott, Janny H Dekker

**Affiliations:** 1 Department of General Practice and Elderly Care Medicine, University Medical Centre Groningen, Groningen, The Netherlands; 2 Department of Medical Microbiology, University Medical Centre Groningen, Groningen, The Netherlands

**Keywords:** *Chlamydia trachomatis*, sexually transmitted diseases, sexual behaviour, women, general practice, primary healthcare

## Abstract

**Background:**

Genital and anorectal *Chlamydia trachomatis* (CT) frequently present together in sexually transmitted infection (STI) clinics.

**Aim:**

To investigate the prevalence of co-occurrent genital and anorectal chlamydia infection, and to study whether sexual behaviour is associated with anorectal infection.

**Design & setting:**

A cross-sectional study in general practices in the north of the Netherlands.

**Method:**

Women attending general practice with an indication for genital chlamydia testing were included and asked to complete a structured questionnaire on sexual behaviour. Anorectal infection prevalence was compared according to testing indications: standard versus experimental (based on questionnaire answers). Variables associated with anorectal chlamydia were analysed by univariate and multivariate logistic regression analyses.

**Results:**

Data could be analysed for 497 of 515 women included. Overall, 17.8% (*n* = 87/490) were positive for CT; of these, 72.4% (*n* = 63/87) had co-occurrent genital and anorectal infection, 13.8% (*n* = 12/87) had genital infection only, and 12.6% (*n* = 11/87) had anorectal infection only. Rectal infection was missed in 69.3% of cases using the standard indication alone, while adding the sexual history still missed 20.0%. Age was the only variable significantly associated with anorectal infection.

**Conclusion:**

The prevalence of anorectal disease is high among women who visit their GP with an indication for genital CT testing. Many anorectal infections are missed despite taking comprehensive sexual histories, meaning that standard treatment of genital infection with azithromycin may result in rectal persistence. Performing anorectal testing in all women with an indication for genital CT testing is, therefore, recommended.

## How this fits in

CT infection continues to be prevalent in women despite screening programmes and awareness among care providers and patients. This could reflect anorectal persistence, with a high rate of infection at both genital and anorectal sites reported in high-risk populations. Data are added from general practice showing a high rate of missed co-occurrent genital and anorectal chlamydia infection with standard diagnosis and treatment. Performing additional anorectal testing for CT in all women with an indication for genital testing is, therefore, advisable.

## Introduction

The prevalence of CT is high and continuing to rise worldwide.^1^ Occurrence of undetected and untreated anorectal CT infection has gained attention as a potential reason for failure to control this epidemic. Testing guidelines for STIs advocate rectal CT screening for women who visit a healthcare facility with a history of anal intercourse or anal symptoms.^2–6^ However, anorectal CT is frequently diagnosed without such a history, as shown in studies of women attending STI clinics or hospitals.^7–9^ One study from an STI clinic in the Netherlands reported a high rate of genital and anorectal CT co-occurrence even with no indication for anorectal testing.^9^ The authors, therefore, concluded that testing based on classical indication is no longer appropriate.^9^ Once identified, rectal infection should be treated with doxycycline because standard treatment with azithromycin, which is used for genital CT in the Netherlands, may fail to clear rectal infection and may promote recurrent genital infection by auto-inoculation.^9^ The British Association for Sexual Health and HIV (BASHH) and NHS guidelines recommend doxycycline as first-line treatment for uncomplicated CT infections.^10,11^ Another problem with anorectal testing by indication is that women might interpret the question 'did you have anal sex?' as meaning anal penetration by the penis, whereas CT may be transmitted to the rectum by other anal contact. To date, studies of anorectal CT in women have been carried out in STI clinics or hospitals where there is typically a high risk of STI.^7^ To the authors' knowledge, there are no data in primary care populations where women opting for an STI test are generally at lower risk.^7^

This study aimed to discover the prevalence of both genital and anorectal CT in women with an indication for genital CT testing when visiting a GP about an STI. It was also aimed to determine if anorectal infection (co-occurrence) could be predicted from an in-depth questionnaire on sexual history.

## Method

### Study design, patients, and setting

This cross-sectional study was conducted in seven general practices with a GP-led STI consultation facility in the north of the Netherlands. These practices delivered protocol-based care following Dutch GP guidelines for patients with STI-related signs, symptoms, or questions. There is no national asymptomatic screening programme for CT. Between September 2017 and October 2019, consecutive women aged ≥18 years with an indication for genital CT testing were informed about the study by a nurse or GP and asked to participate. The following indications were used: multiple sexual partners in the past 6 months; unprotected sex; a sexual partner with a STI; vaginal symptoms; and fear of having an STI. Women were excluded if they refused anorectal testing or were unable or unwilling to provide an adequate sexual history.

### Data collection

A sexual history was obtained using a structured questionnaire administered by the nurse or GP (Supplementary Figure 1). Standard questions were first asked about anal sex and symptoms. Thereafter, extended questions were asked about anal contact with and without penetration, condom use for anal sex, anal contact with fingers, use of sex toys, and oral contact with either the patient’s genitals or anus.

Genital and anorectal samples were taken for CT testing after receiving informed consent from the patient. Self-collection at home was allowed, for which clear written and verbal instructions were given on how to take the samples (for example, to prevent cross-contamination). The swabs were sent to a laboratory (Certe, Groningen) for CT testing by real-time polymerase chain reaction (PCR). Deoxyribonucleic acid (DNA) was isolated from samples, using MagNAPure 96 (Roche Diagnostics, Germany), according to the manufacturer’s protocol, and was tested using the Presto CT-NG assay (Goffin Molecular Technologies). The PCR cycle threshold (Ct) value was recorded for all positive samples, with lower Ct values indicating larger amounts of DNA (that is, inversely proportional).

To facilitate comparison with other studies, patients were grouped into three age categories and the indication for testing was based on data from the sexual behaviour questionnaire. 'Standard' indications for anorectal testing were considered as self-report of anal symptoms and/or anal sex, and 'experimental' indications for anorectal testing were considered to be any positive answer to at least one relevant question from among all standard and extended questions on anal contact.

Demographic data and questionnaire responses were recorded on study forms by the GPs and nurses of the general practice and sent to the study centre after anonymisation. All data were imported in an Excel database and independently checked for input errors. Patients were treated according to the Dutch guideline on STI in general practice, based on their test results (azithromycin for genital CT and doxycycline for anorectal or double infection).

### Sample size

It was expected that 10% of the genital CT tests would be positive based on results from a previous study,^12^ and it was assumed that, among these, 50% of subsequent anorectal tests would be positive. Therefore, given a population of 500 tested women, it was expected that genital CT would be present in 50 (95% confidence interval [CI] = 37 to 63), with anorectal CT present in 25 of these (*n* = 25/500 = 5%; 95% CI = 3% to 7%). This was considered sufficiently accurate for the study aims.

### Statistical analysis

The two standard and experimental indication categories were analysed for their ability to predict anorectal CT infection. Missed anal infections were categorised as 'missed by standard indication' (no anal sex or anal symptoms, but a positive anorectal test) and 'missed by experimental indication' (no anal sex or symptoms, no positive response to questions in the extended history, but a positive anorectal test). To describe the anatomic distribution of CT-positive cases, patients were grouped into non-overlapping categories (genital only, anorectal only, or anorectal and genital). Descriptive data are presented as medians, ranges, and interquartile ranges (IQRs). Univariate and multivariate logistic regression were used to identify variables associated with anorectal CT. Univariate analysis included age, standard indication, experimental indication, and each questionnaire item separately. Variables with a significant association (*P*<0.05) were then included in a multivariate logistic regression model. A stepwise backward-elimination selection strategy was followed to arrive at a model that included only the predictors with *P*<0.05, reporting their odds ratios (ORs) and 95% CIs. In a post-hoc analysis, the correlation between anorectal and genital Ct values were explored. All analyses were performed using IBM SPSS (version 23.0).

## Results

### Participants and descriptive data

In total, 515 patients from seven general practices were included by 16 practice nurses and four GPs. Only a few eligible women refused to participate, but neither the actual number nor the reason were recorded (anecdotally, most felt uncomfortable having an anorectal swab taken). In addition, data were excluded for 17 patients because their rectal tests were taken 2–9 days after the genital test; retest data from one patient was excluded after a previous positive test. This left 497 patients for the analyses. Both anorectal and genital CT test results were missing in seven patients (most likely because participants had not sent their self-collected samples to the laboratory); only the anorectal test was missing in three cases; and only the genital test was missing in another three cases. Therefore, both rectal and genital CT test results were available for 484 participants. Fewer than 1.5% of the questionnaires had missing values, so imputation for missing data was not performed.

Table 1 shows the distribution of patients by age and indication for rectal CT testing, together with the prevalence of CT by site. The median participant age was 25 years (range, 18–72; IQR, 22–30) and the overall prevalence of CT (urogenital and/or anorectal) was 17.8% (*n* = 87/490). Of the CT positives, 72.4% (*n* = 63/87) had a double infection, 13.8% (*n* = 12/87) had a genital mono-infection, and 12.6% (*n* = 11/87) had an anorectal mono-infection. In total, 42.5% and 83.3% had an indication for anorectal testing according to the standard and experimental indications, respectively. In the experimental indication group, 14.8% (*n* = 60/406) tested positive, whereas 18.5% (*n* = 15/81) of the remaining women tested positive (OR: 0.76; 95% CI = 0.41 to 1.42; *P* = 0.40). Of the positive cases, 69.3% (*n* = 52/75) were in the group with no indication for anorectal testing according to the standard guidelines and 20.0% (*n* = 15/75) were still missed.

**Table 1.** Population characteristics and *Chlamydia trachomatis* prevalence by indication for rectal testing

	Standard indication*N* = 211^a^% (*n/N*)		Experimental indication*N* = 414^b^% (*n/N*)		Total*N* = 497% (*n/N*)	
**Age, years**						
≤21	15.6	(33/211)	20.0	(83/414)	21.7	(108/497)
22–28	44.1	(93/211)	46.4	(192/414)	45.1	(224/497)
>28	40.3	(85/211)	33.6	(139/414)	33.2	(165/497)
Chlamydia prevalence^c^						
Any site	12.0	(25/208)	16.7	(68/408)	17.8	(87/490)
Urogenital	10.1	(21/207)	14.5	(59/406)	15.4	(75/487)
Anorectal	11.1	(23/208)	14.8	(60/406)	15.4	(75/487)

^a^One missing indication owing to missing questionnaire data. ^b^Three missing experimental indication owing to missing questionnaire data.^c^Denominators were adjusted to the number of participants tested at each site (any, urogenital, or rectal). Both tests were missing for seven, of which three had a urogenital test only (all negative) and three had a rectal test only (one was positive).

In the group defined by standard, anal symptoms were reported by 20.0% of patients and anal sex by 28.2%, with both reported in 5.7% (Table 2). The most common anal or bowel symptoms were itching (*n* = 43), bleeding (*n* = 38), pain (*n* = 23), haemorrhoids (*n* = 16), burning sensation (*n* = 12), and discharge (*n* = 11). Some patients also reported redness (*n* = 6), irritable bowel syndrome (*n* = 6), diarrhoea (*n* = 5), constipation (*n* = 5), and anal fissure (*n* = 5), and there were single reports of swollen anus, dry skin, proctitis, irritation, ulceration, and irregular stool. Symptoms also frequently occurred together. According to the answers on the structured sexual history questionnaire, anal sex was reported without penetration by 47.7% and with penetration by 21.1%. Anal contact with fingers was reported by 38.4% and with toys by 7.1%. Oral contact with the genitals was reported by 72.6% and oral contact with the anus by 18.5%.

**Table 2.** Results of the questions regarding anal sex, symptoms, and sexual behaviour in the past 6 months

Indications	%	*n/N*
History according to standard guidelines		
Anal symptoms	20.0%	(99/495)
Anal sex	28.2%	(140/497)
Anal sex and anal symptoms	5.7%	(28/495)
Total indications for anorectal testing (anal sex or symptoms)	42.5%	(211/497)
Additional sexual history		
Anal sex without penetration	47.7% (3.3% condom use)	(236/495)
Anal sex with penetration	21.1% (13.7% condom use)	(104/492)
Anal contact with fingers of partner	38.4%	(189/492)
Anal contact with toys	7.1%	(35/492)
Oral contact with genitals of the woman	72.6%	(358/493)
Oral contact with anus of the woman	18.5%	(91/493)
Resulting experimental indication for anorectal testing (positive answer on any anal contact)	83.3%	(414/497)

*n* is the total number positive answers for the variable, *N* is the total number of women who answered that question (excluding the missing answers).

### Variables associated with anorectal CT infection

Women with a standard indication for anorectal testing had significantly less anorectal CT. Participants reporting anal sex with penetration, anal contact with fingers, or oral—anal contact had significantly lower anorectal CT rates than those answering 'no or do not know' on these sexual history questions (see Table 3). Age was significantly associated with anorectal CT, with infection more common in younger patients. Multivariate logistic regression including these four variables resulted in a significantly independent association only for age: OR 0.40 (95% CI = 0.23 to 0.71; *P* = 0.002) for the age group 22–28 years and OR 0.20 (95% CI = 0.09 to 0.41; *P*<0.001) for age group >28 years when compared with the age group ≤21 years.

**Table 3.** Anorectal chlamydia and association with different variables

Variables	*n*	Rectal CT(%)	Univariate analysisOR (95 %CI)	*P* value	Multivariate analysisOR (95% CI)	*P* value
Standard indication for rectal testing	208	(11.1)	0.54 (0.32 to 0.92)	0.023*		
Experimental indication for rectal testing	406	(14.8)	0.76 (0.41 to 1.42)	0.40		
Anal symptoms	98	(12.2)	0.72 (0.37 to 1.39)	0.35		
Anal sex	138	(10.9)	0.59 (0.32 to 1.07)	0.10		
Anal sex without penetration	230	(12.6)	0.67 (0.41 to 1.12)	0.13		
Anal sex with penetration	103	(7.8)	0.40 (0.19 to 0.86)	0.020*	0.64 (0.28 to 1.47)	0.29
Anal contact with fingers	185	(9.2)	0.43 (0.24 to 0.76)	0.003*	0.58 (0.31 to 1.11)	0.10
Anal contact with toys	34	(5.9)	0.33 (0.08 to 1.42)	0.14		
Oral contact with genitals	352	(15.6)	1.09 (0.62 to 1.92)	0.89		
Oral contact with anus	91	(7.7)	0.40 (0.18 to 0.91)	0.024*	0.69 (0.29 to 1.66)	0.41
Age, years^a^						
≤21	104	(30.8)				
22–28	220	(14.5)	0.38 (0.22 to 0.67)	0.001*	0.40 (0.23 to 0.71)	0.002*
>28	163	(6.7)	0.16 (0.08 to 0.34)	<0.001*	0.20 (0.09 to 0.41)	<0.001*

^a^Age ≤21 years was the reference category. *n* is the total number positive for the variable, excluding 10 women without a rectal CT test. The rectal CT positive (%) was calculated by excluding women with a missing answer (0–5 per question). Women answering 'don’t know' were added to the 'no' category when calculating odds ratios and Fisher’s exact *P* values. Significant *P* values ≤0.05 are indicated by asterisk (*). Only possible risk-factor variables (below the double line) were analysed with logistic regression if significantly associated with rectal CT in the univariate analysis. CT = *Chlamydia trachomatis*

### Anorectal and genital bacterial load by cycle threshold values

**Figure 1.**
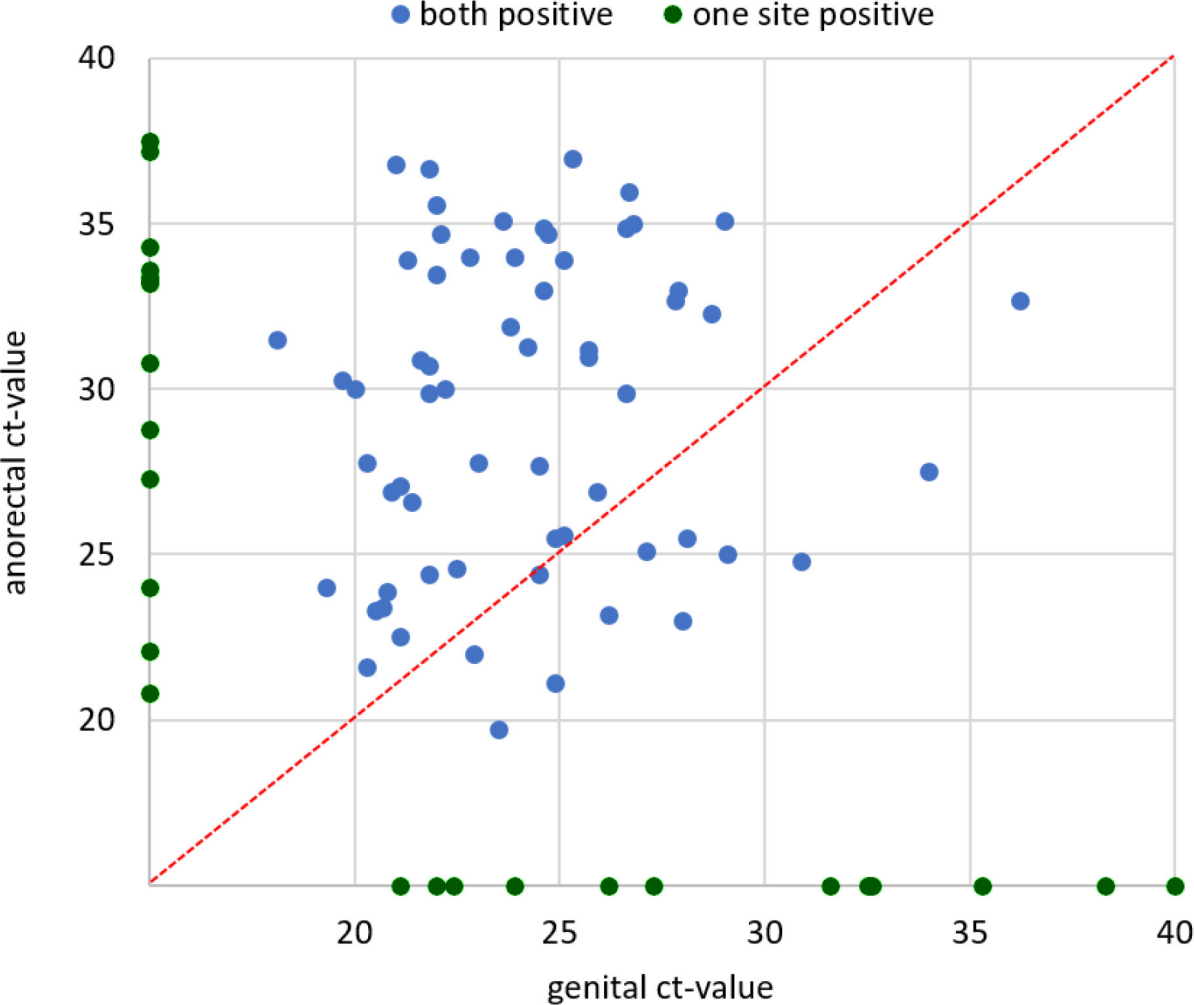
Distribution of cycle threshold (Ct) values of samples positive in the *Chlamydia trachomatis* polymerase chain reaction (PCR) test

Ct values were available for 86 PCR-positive cases. Genital and anorectal Ct values were missing for one woman with a positive PCR test. The median Ct values for positive PCR tests were 24.4 (range, 18.1–40.0; IQR, 22.8–26.9) and 30.2 (range, 19.7–37.5; IQR, 25.0–33.9) in the genital and anorectal samples, respectively. Ct values did not differ by age (data not shown). Of the 62 patients with double infection and Ct values for both test sites, the genital Ct value was lower (indicating a higher bacterial load) in 50 (80.6%). Figure 1 shows the distribution of Ct values.

## Discussion

### Summary

The study found a high prevalence of urogenital and anorectal CT among women visiting their GP with STI-related symptoms or questions and for whom a urogenital CT test was indicated, with almost three-quarters having infection at both sites. Younger age was associated with higher rates of anorectal CT, and self-reported anal sex or symptoms (that is, the basis for standard anorectal testing) were associated with less anorectal CT. After multivariate logistic regression, only age retained a significant association, and this was not affected by expanding the standard indication for testing to include questions on sexual behaviour. The proposed experimental indication also missed a fifth of the anorectal infections, despite most of the study population being in this group.

### Strengths and limitations

The results of 10 urogenital and 10 rectal CT tests were missing for samples collected at home. Although participants could not be contacted to ask why, loss of interest or fear of taking a sample are plausible reasons. Participation in research may also have led to patients reporting more anal symptoms than would normally be the case, leading to an artificially higher prevalence than normally found in clinics. Moreover, condom use was only asked about if anal sex was reported, including no questions about prior STI or contact notification. Finally, the study was not powered to detect associations between determinants and actual anorectal CT.

The number of variables in the logistic regression analysis was set to a minimum of 10 cases per independent variable added to the model. Although this is a standard approach, analyses of variables with a low prevalence (for example, anal contact with toys) will produce results that have limited power and must be interpreted with caution. However, the exploratory results support those reported by van Liere *et al*,^9^ indicating that many anorectal infections will be missed when testing based on sexual technique alone.

Despite the authors' best efforts to prevent cross-contamination of anorectal and genital samples, they cannot be certain that self-collected samples were not contaminated. CT levels were certainly lower for anorectal samples than for genital samples (higher Ct values), but direct comparison is not possible given how much the different sample sites affect bacterial load.^13^ A significant proportion of the anorectal tests also had Ct values consistent with at least moderate DNA loads, arguing against contamination as the only explanation for its presence on rectal swabs. Furthermore, it is not known whether high Ct values indicate contamination or inactive infection with low transmissibility. These possibilities should be addressed in future research.

Major strengths of this study are that it was carried out in general practice, with a large sample, and with almost all eligible women. These features contribute to the generalisability of the results to primary care settings in The Netherlands. Furthermore, detailed questions were asked on sexual behaviour that may be associated with anorectal CT. This strengthens the conclusion that anorectal CT cannot reliably be predicted in women based on sexual behaviour or symptoms alone.

### Comparison with existing literature

The prevalence of CT in the study was higher than previously reported in Dutch primary care (10%–11%),^9^ and was closer to that reported in sexual health centres (15%).^12^ In England, a CT screening programme reported a positivity rate of 10% between 2018 and 2019.^14^ The authors' assumption that women visiting their GP for STI-related symptoms or questions would have a lower risk of STI than women visiting STI clinics might not be correct. Practices were only included where a structured STI consultation was done by a practice nurse, possibly leading to bias because they may have more frequent consultations for STI than other practices. Although this could reflect location, such as areas with high-risk populations, only three of the participating practices were located in the inner city of Groningen and the other four were located in more rural areas. The percentage of anorectal infections that were missed when testing based on the standard indication (69.3%) was comparable with that in an STI clinic (70.9%).^9^ Standard guidelines do not detail specific anal symptoms and compared with previous research in an STI clinic, the present study found that symptoms were more varied.^9^ However, a relationship with CT infection was not plausible for all of these symptoms.

### Implications for research and practice

A comprehensive sexual history does not help to identify women who require anorectal testing, and as such, cannot be recommended. Instead, it is recommended that case identification should be improved by offering anorectal tests to all women with an indication for genital CT testing. An alternative may be to treat all positive urogenital CT tests with doxycycline, as is already recommended in BASHH and NHS guidelines, and omit rectal testing entirely. That is a cheaper strategy than double testing, but it risks leaving anorectal mono-infection untreated. Also, there needs to be an understanding on how such an approach affects the prevalence of CT.
